# Undervalued professionals: placement of nutritionist in the Ecuadorian health system

**DOI:** 10.1186/s12913-023-09340-8

**Published:** 2023-04-26

**Authors:** A. C. Román, M. A. Villar, P. Belmont-Guerron, M. B. Ocampo

**Affiliations:** 1 Colegio de Ciencias de la Salud, Nutrición y Dietética, Universidad San Francisco de Quito USFQ, Campus Cumbaya, edificio Hanna Arent, oficina HA102, Casilla Postal, 17-1200-841, 170901 Quito, Ecuador; 2 Escuela Politécnica Nacional, Facultad de Ingenieria en Sistemas, Quito, Ecuador

**Keywords:** Nutritionist, Clinical nutrition, Health system, Work conditions, Dietetic practice, Public health professionals

## Abstract

**Background:**

Currently, many public health issues are directly related to malnutrition, and are made worse by social inequities. Nutrition professionals must be a key player in improving epidemiological aspects of nutrition-related diseases and must be part of clinical teams to control nutritional concerns.

**Objective:**

To identify and analyze the nutritionists´ employment situation in Ecuador and areas of work covered and determine if type of university has an impact over work situation.

**Methodology:**

A cross-sectional study was conducted, approved by the ethics committee of Universidad San Francisco de Quito. It included 442 nutritionists in Ecuador who graduated in 13 universities (5 private (PR) and 8 public (PU)) between 2008 and 2019. It implied an online survey that questioned their satisfaction with their education and current work situation. All the statistical analyses were performed using R version 4.0.3, two-sided weighted chi-square test was performed to estimate the difference between public and private university graduates, IC 95%, ***p*** between 0.01 and 0.05.

**Results:**

38,6% of participants are unemployed, 68,28% private university graduates (PR) are currently employed and 58.87% work as nutritionists, compared to 56,86% from a public university (PU) currently working and 44.69% working in the field. 76% have reported being unemployed at some point in their careers, being difficulty finding jobs the main reason. Regarding the professional field, most professionals have their own business, and the less common area of work was public and community nutrition. One third of the participants had another paid activity. The main salary is 800USD per month and graduated from PR perceived better salaries than from PU.

**Conclusion:**

There is a lack of job opportunities for Ecuadorian nutritionists despite the high demand in every level of the health system. Most have been unemployed at some point in their careers due to difficulties finding jobs. There is a minimum nutrition staff working in community and public health nutrition.

## Background

In Ecuador there are many nutritional concerns that should be addressed by professionals in the nutrition and dietetics area, some of these public health issues result from malnutrition. Some of the main public health issues in Ecuador result from malnutrition. In 2018, 63.6% of adults had some degree of overweight and obesity, 19,8% of adults had hypertension, 12.3% of older adults suffered from type 2 diabetes, 34.7% of adults had high total cholesterol [1]. Additionally, Ecuador has the second highest rates of chronic malnutrition in Latin America, representing a total of 23% of children under 5 years and 27.2% of children under 2 years of age [2]. Most nations are undergoing a nutritional epidemiologic transition, causing populations to suffer from chronic diseases or lack of sufficient access to foods [3].

Considering the high prevalence of nutritional-based public health concerns in Ecuador, made worse by social determinants such as food insecurity, government policies, health inequalities such as lack of basic services, poverty, non-universal health coverage and lack of health promotion services [4]; nutrition professionals must be a key player in improving not only epidemiological aspects of nutrition-related diseases, but also involved in enhancing access to social, health and basic services. Thus, having professionals with an academic background in nutrition in the public health field constitutes a promising resource for the development of action plans to eradicate these nutritional concerns [5].

The presence nutritionists in the healthcare system is necessary to address relevant clinical concerns, improve patient outcomes [6], and solve important public health issues. Nevertheless, there are two main barriers that keep nutritionists out of multiactor and multisectorial teams.

First, in Ecuador, undergraduate and graduate training in nutrition has had historical curricular differences. Nowadays, sixteen public and private universities are offering this program, and until five years ago, each university worked with different pensums and offered distinct professional titles in nutrition [7]. These differences could determine the ability of graduates to find paying jobs after graduation, the rate of unemployment, higher job satisfaction, and application of specialized skills and knowledge on the job. Second, the current organization of the Ecuadorian public healthcare system does not include nutrition services in every level of care [8], which should be the frontline for preventing non-communicable diseases and chronic malnutrition.

Dealing with diet-related diseases should include a multidisciplinary approach that enables addressing different issues besides consumption, from the initial stages of food production until the final distribution but also economical, marketing, and environmental issues [9]. Undoubtedly, nutritionists must be part of this multidisciplinary team, due to their academic background and knowledge regarding diet-based diseases, and capability of proposing prevention and treatment options. However, in Ecuador, public information regarding the job occupation and satisfaction of nutritionists in Ecuador is currently unknown. Therefore, the purpose of this study is to answer the question about nutritionists´ employment situation in Ecuador and areas of work covered, additionally, address if there are differences in these rates depending on the type of university where they graduated.

## Methodology

### Study design

The cross-sectional study encompasses all nutritionists in Ecuador who graduated between 2008 and 2018 selected using stratified random sampling design. It implied an online survey that questioned their satisfaction with their education and current work situation. The survey was conducted by phone, from November 2019 to April 2020. Questionnaires were applied by two trained nutritionists supervised by the main researchers. All participants had to sign an online consent form before answering the survey, and data collected using an online survey repository. The ethics committee of Universidad San Francisco de Quito reviewed and approved this study. (COD 2019-003IN). STROBE checklist was exhaustively considered throughout the methodology and completed accordingly in the text [10].

### Data collection

An online survey questioned nutritionists satisfaction with their education and current work situation. Study participants were recruited by randomly selecting individuals from a complete list of 2498 nutritionists based on graduates from each participating university. Data was collected by interviewers sending out a text message inviting nutritionists to participate in the study. After receiving 2 weekly text message reminders, and if the survey was not yet complete, interviewers collected the data through phone calls in an online survey repository using Open Data Kit (ODK) survey technology. The survey was applied a single time to each participant.

### Participants

In total, 13 universities (5 private and 8 public) with an undergraduate nutrition program were identified using administrative records from every institution. No records of graduates with Licensed Nutritionist as a bachelor’s degree were held before 2008. An exhaustive list of professional nutritionists was compiled, based on the yearly graduate rate per university between 2008 and 2018. Nutritionists outside this period of graduation were excluded. Participant were first contacted by text message randomly selected from list until completing sampling requirements. Participants not working in the field of nutrition at the time of the survey, but with an undergraduate degree in nutrition, were also included.

### Questionnaire

The questionnaire consisted of 38-items, measuring job satisfaction, job opportunity, and professional recognition perceived by nutritionists. A second part focused on the perceived importance of scientific knowledge and skills as practitioners. The questions included in the survey were taken from two studies carried out in Mexican nutritionists [11, 12], and the questionnaire was constructed by the authors. Table 1 shows the outcomes measured through multiple choice and Likert scale questions.

**Table 1:** Variables measured by questionnaire

Variable	Type	Description
**General Characteristics**		
Region	Categorical	Region
University	Categorical	University
Sex	Binomial	Sex
Age	Categorical	Age
Civil status	Categorical	Civil status
Status	Categorical	Employment status
Unemployment	Binomial	Ever unemployed
Unemployment motive	Categorical	Reason of unemployment
Unemployment period	Categorical	Maximum period of unemployment
Graduation Year	Numerical	Graduation year
Field	Binomial	Work in the nutrition domain
Field nutrition	Categorical	Field of nutrition
Field reason	Categorical	If not working in the nutrition field, reason to abandon the nutrition field
Period until first job	Categorical	Elapsed time until first job after graduation
**Knowledge and skills**		
Pensum_satisfaction	Likert	Pensum satisfaction related to professional practice
Pensum_quality_perception	Likert	Pensum quality perceived by nutritionists
Knowledge difficulties	Binomial	Has the nutritionists perceived difficulties because of poor knowledge impaired in university
Knowledge principal	Categorical	Most important knowledge selected in list
Knowledge satisfaction	Likert	Of specific impaired knowledge, how satisfied is nutritionists
Skills requiered	Categorical	Skills requiered as professional
Skill principal	Categorical	Most important skills selected in list
Skills satisfaction	Likert	Of specific impaired skills, how satisfied is nutritionists
**University degree**		
University degree	Categorical	Education level
Course modality	Categorical	Course modality
Postgraduate satisfaction	Likert	Curriculum in postgraduate satisfaction
Postgraduate country	Categorical	Country of postgraduate degree
**Work environment**		
Workload	Numerical	Daily hours dedicated to the first activity
Salary range	Categorical	Salary range
Salary fixed	Binomial	Is salary fixed or variable
Secondary activity	Binomial	More than one job
Secondary activity field	Categorical	Field of nutrition of the second activity
Nutritionist role satisfaction	Likert	Perceived Satisfaction of the role of Nutritionist
Salary satisfaction	Likert	Perceived Satisfaction of the salary
Work satisfaction	Likert	Perceived Satisfaction of the current job
Work rise possibility satisfaction	Likert	Perceived Satisfaction of the rise possibility
Work environment satisfaction	Likert	Perceived Satisfaction of the work environment

### Sampling procedure

To avoid selection bias, participants were selected randomly, through a probabilistic random stratified sample design, using type of university (public or private) as strata, and yielding a sample size of 442 individuals, using: n = z² * p *q / c²* FPC Where: z:confidence level of 95% (z = 1.96), p: a variance (50%) and c error rate (6%), FPC: finite sample correction: FPC = ((N-n)/(N-1))1/2 Where: N = population size, n = sample size.

The response rate was around 56%, common for this type of contact, but did not impact quality of results or sampling. A total of 442 valid responses were collected. Expanded data allowed to obtain estimates separated by university types.

### Analysis

All the statistical analyses were performed using R version 4.0.3 (R Core Team, R Foundation for Statistical Computing, Vienna, Austria, 2020). Categorical variables were codified when categories summed less than 8 observations. Considering sampling effect and stratification, a two-sided weighted chi-square test was performed to estimate the difference between public and private university graduates, reporting obtained p-value for a confidence level of 95% and significance for value of p between 0.01 and 0.05. Frequency of missing data was negligible, sampling weights were calculated and considered for all statistical tests.

## Results

The response rate was around 56%, even though nutritionists accepted to participate through text message, they didn´t complete the survey because of lack of time and job obligations.

Demographic characteristics of the 442 participants are shown in Table 2. To remove bias, this study included a proportional number of participants that attended a private university (41.86%) and a public university (58.14%). Consequently, the further expanded data provides estimates according to university types. 84,2% of the participants were women an 74,9% were not married. 50,63% of the participants currently work as nutritionist, while 11,01% don’t, and the remaining 38,36% report being unemployed at the time of the survey.

**Table 2:** Licensed Nutritionist personal characteristics (442)

Characteristics		n	%
Gender	Female	381	84,2
	Male	61	15,8
Age	20–25 years	93	19,7
	25–30 years	234	52,6
	30–35 years	104	24,1
	35 or more years	11	3,6
Marital status	Married/Partnered	104	25,1
	Single/Not married	338	74,9
Year of graduation	2008–2014	114	30
	2015–2019	328	70
Year since graduation	1–2 years	232	47,2
	3–11 years	210	52,8
University type	Private	183	41,9
	Public	259	58,1
Region	Coast	170	40
	Sierra	272	60
Postgraduate	Yes	165	37
	No	277	63
Practicing as nutritionist	No	50	11
	Unemployed	179	38,4
	Yes	213	51
Total		442	100

Table 3 describes employment status by university type. Data was analyzed based on the affiliation of participants to public or private universities, to have a better picture of how curriculums were organized and implemented in each area, and their influence in training of nutritionists. There is a significant difference between the employment status of participants that graduated from a private university (PR) in which 68,28% are currently employed and 58.87% work as nutritionists, compared to 56,86% from a public university (PU) currently working and 44.69% working in the field.

When participants were asked about being unemployed at some point in their professional life, 76% reported being in this situation at least once. There were no statistical differences between socio economic groups in this variable, however male participants reported less time without a job than female (up to 12 months 28%, and 38% respectively). When asked about the reason of ever being unemployed, 70% of nutritionists referred difficulties finding a job as the main reason, 46% referred to low salaries and 12% to a non-convenient work schedule. 51.46% of PR vs. 40.97% of PU ( (p = 0.033) nutritionist perceived finding a job as the main reason for ever being unemployed. When asked about how long it took to find a job after graduation, 44% of PR graduates and 31% of PU reported finding a job immediately, while 42% of PU and 41% from PR graduates took between 6 and 12 months after graduation to be hired.

**Table 3:** Current job status by university type (442)

Characteristics			University type				
			Private		Public		
			n	%	n	%	p-value
Employment status	Currently working	No	57	31,8	122	43,1	0.027 *
		Yes	126	68,3	137	56,9	
	Work as nutritionist by type of university	No	16	9,4	34	12,1	0.021 *
		Unemployed	57	31,8	122	43,1	
		Yes	110	58,9	103	44,7	
Total		n	183	100	259	100	

* Significance for value of p between 0.01–0.05

**Fig. 1:**
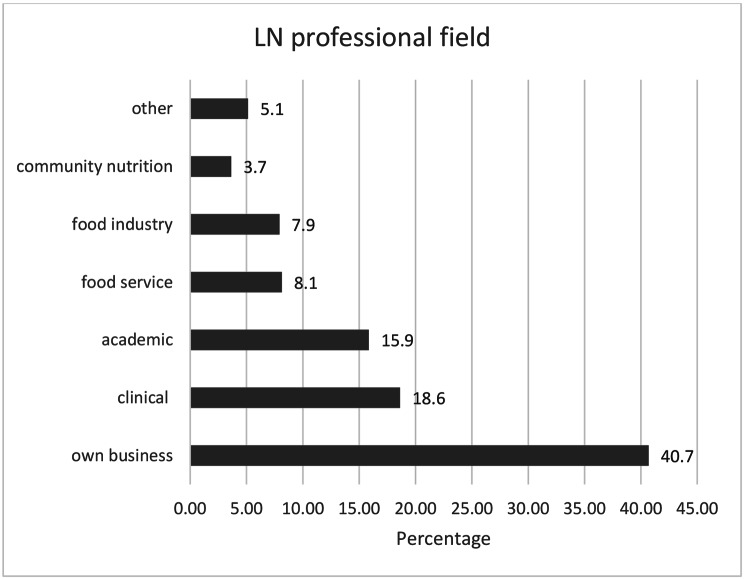
Nutritionists professional field distribution

Results in Fig. 1 showed that most professionals have their own business (40.69%), while the least common field of work is community nutrition (3.64%). The main activity of PR graduates was clinical nutrition (23.3%) compared to 15,9% from PU (< p = 0,05). Of those who stop working in the field, 28.9% reported economic issues as the main problem and the other 67.7% did not specify the reason. One third (28.6%) worked in a second economic activity, 15,8% more of PR graduates had another activity (PR 37.2% and PU 21.4%).

Regarding work conditions (Table 4), the predominant nutritionists’ income was 800 USD per month for both PR (34.93%) and PU (56.71%) graduates (p = < 0,01). 54,39% of PR earned over 1000 USD income compared to 30,92% from PU. No participants from PU reported an income of 2500 USD. 64,34% of nutritionists from PU have a stable income, as compared to 43,1% from PR.

**Table 4:** Nutritionists´ work conditions by university type

Characteristics		University type					
		Private			Public		
		n			n		
		sum	Percent	p-value	sum	Percent	p-value
Income type	Fixed	41	43	0,442	79	64,3	0,442
	Variable	27	18		47	35,7	
Work hours/day	4	12	8,8	0,413	17	13,8	0,413
	6	23	15,9		17	12,8	
	8	75	75,3		92	73,4	
Nutritionists income (USD)	800	40	34,9	0.001 ***	77	56,7	0.001 ***
	800–1000	12	10,7		11	12,4	
	1000–1500	35	34,6		30	21,4	
	1500–2500	15	12,6		8	9,5	
	2500	8	7,2		0	0	
Total		110	10		126	100	

* Significance for value of p between 0.01–0.05

## Discussion

There are limited job opportunities for nutritionists in Ecuador, which is contradictory to the fact that there are approximately 250 to 300 hundred new nutrition graduates per year. Shown by the fact that 38,36% report currently being unemployed. 44.75% of all participants stated that the reason for unemployment at some point in their careers was an inability to find a job. Currently, 5.1% of the Ecuadorian population is unemployed; of those that do not have a job, 24.9% are young adults that have recently graduated and are looking to begin their professional careers [13, 14].

A total of 84.2% of participants were female, showing that it is a female-represented profession. According, the Gran Encuesta Integrada de Hogares de Colombia 2019 (GEIH) there are 8% more women than men in the healthcare system, specifically female doctors, dentist, pharmacist, nurses and nutritionist [15]. In Latin America, some professions like nursing and nutrition are female-dominated profession, for instance in Colombia 89% o nurses and 92.7% of dietitians are women [15]. The same as in Chile, where women occupy 95% of obstetrician jobs, 86% of nursing and 90,6% of nutrition jobs [16]. In Canada, over 95% of nutrition jobs are taken by women [17]. The United Nations Development Program (UNDP) describes that, despite being a feminized job, women receive 29% less income and less professional value than men [18].

It is also important to explore how men feel in this profession. Gheller et al., (2018) conducted semi-structured interviews to male nutritionists and established the feelings of difference and otherness, gender stereotypes during training and into their career, low pay and low prestige if compared to other healthcare professions, as the main reasons for men underrepresentation [19]. Joy et al., (2019), indicated that the promotion of certain gender beliefs such as women´s domain in food areas will perpetuate division and differences within this profession [20].

There are two main points to analyze, the occupational situation and if there are differences in job opportunities because of training curriculums.

First, the results obtained in this study give an insight into the scarce job opportunity for nutritionists in Ecuador. Regarding their professional field, most nutritionists own their business-related or not to nutrition (40.77% PU vs. 58.28% PR). This result emphasizes the scarce supply of jobs in the country for nutritionists, since a considerable percentage resort to entrepreneurship, instead of working in the already existing academic, food services, food industry or community nutrition fields. The lack of professionals in the community nutrition field raises another issue. The International confederation of Dietetic Associations, states that in 2016, 86% of the member countries (42) have nutritionists working in the public health area while 100% reported hospitals as location of employment either as clinical dietitians or food services/hospitality [21]. The fact that the results showed that less than 3% of Ecuadorian nutritionists work in this field is alarming. The Colombian Association of Nutrition and Dietetic Faculties (ACOFANUD) reported that 43% of Colombian dietitians worked in public health areas follow by clinical nutrition (30%) [22]. The current organization of the Ecuadorian public healthcare system does not include nutrition services in every level of care, furthermore emphasizing the scarce work opportunities for nutritionists [8]. Nutritionists working in the public sector provide services only in healthcare places belonging to the second level of care, more specifically in day hospitals and specialized hospitals. First level of care centers in the public sector do not provide nutritional care services because that specific position has not been contemplated [23]. The fact that there are none on this level, not only reduces work opportunities, but also limits nutrition education received by patients.

Furthermore, in this study, hospitals are the second field in which nutritionists are employed in (26.18% PR and 15.95% PU). According to the president of the National Association of Nutritionists in Ecuador (ANNE), there are two – full-time (40 h per week) nutritionists per 200 beds in hospitals [24]. Their job is not only to be in charge of inpatient units but also hospital catering and general practice. Moreover, no regulation determines that a hospital under 40 beds has to have a nutritionists [24].There is not any normative to standardize the number of nutritionists that should be hired in Ecuador. In Chile, Crovetto (2015), determined that there is an important nutrition resource deficit, 43–57% deficit in minimum to medium complexity hospitals (< 299 meals) and 46% in high complexity hospitals (> 300 meals) [25].

According to the Health Ministry of Peru, for each hospital to provide adequate nutritional care, there should be a minimum 1 nutritionists per 40 beds of intermediate care patients, 1 per 15 intensive care patients, and 1 per 15 pediatrics patients. Moreover, in the food production areas there should be also 3 nutritionists per 40 beds, 1 to plan all meals, 1 to supervise production and 1 to supervise the distribution of food [26]. The normative in Chile established 1 nutritionist 20–41 beds depending on the specialty of the Service [27]. Nevertheless, in 2015, Crovetto determined a 57% of deficit of nutritionists in Chilean hospitals. There is no official data from the Ecuadorian Ministry of Health on this subject, thus making it a substantial comparison problem. The Ontario Clinical Nutrition Leaders Action Group (CNLAG) reported a staffing level ratio of 2.0 registered dietitians (RD) working full hours per 100 beds, ranging from 1.0 to 5.0 RD per hospital [28]. Moreover, staffing levels have been recognized as a relevant indicator for patient safety [29]. The Association of UK Dietitians reported that 57% of NHS dietetics staff felt their caseload is manageable, but 60% of these felt they were working overtime. The remaining 43% who felt their caseload was not manageable worked overtime and just 3 of 9 were paid for the additional hours [30].

A total of 74% of nutritionists work 40 h per week, 64% from PU and 43% from PR had stable jobs with law benefits such as social security. Mostly PU graduates earned up to 9.600 USD a year and PR graduates had the same percentage earning up to 9.600 USD (34.9%) to 18,000 per year (34.6%).

Those that get a job in the public healthcare system receive a monthly salary of $901-1,212 ($12,000–16,000 yearly) [31]. This is much lower than the median pays for RD in the United States, which adds up to $63,000 per year [32]. According to the Department of Labor of U.S, to work as RD in the United States it is not mandatory to have work experience because training is complete in an internship and residency. Nonetheless, several participants implied that the biggest issue to apply for a job in the public system in Ecuador is the requirement of 3 to 5 years of experience and a fourth-degree certification (Masters or PhD). This is inconsistent with the fact that 37% of the participants have obtained a master’s degree, 16.57% PU and 18% PR. Mst of these participants studied as soon as they finished their career, so job opportunities do not arise because of the lack of experience. It is important to notice that the differences in salary could obey the year of experience.

Second, this study analyzed the differences between graduates from PR an PU. Nutrition and Dietetics started as a professional degree in Ecuador in 1972 [33], but it wasn’t until 2018 that the Ecuadorian government dictated a curriculum unification to promote equivalent qualifications between nutrition professionals across all schools. Despite some differences between PR and PU graduates concerning employment and salaries there is not enough data to assume that the school attended is a determining factor for professional placement.

## Limitations

Some data are estimated and non-official because information about this subject is lacking. In fact, when the nutrition faculties were asked about information about graduates more than half refused to give any. Self-absorption of information is a limitation for research in Ecuador. Additionally, there is a high sense of rivalry between Ecuadorian universities, which hinders the data collection process.

Another limitation arose from the questionnaire itself, because it allowed the participants to exit the survey after completing specific questions, depending on their answer choices, ending up with incomplete results.

## Conclusion

This study provides information about the lack of job opportunities for Ecuadorian nutritionists despite the high demand in every level of the health system, similar situation various Latin American countries. In Ecuador sixteen nutrition schools keep graduating professionals into a health system that is not willing to open more job positions to create job opportunities. There is a high unemployment rate for nutritionists and an elevated dropout rate from the profession. Furthermore, it is still a profession dominated by women and the most important finding is the lack of nutrition staff working in community and public health nutrition.

## Implications for research and practice

The importance of nutritionists in the health system has increased. Consequently, the necessity of having nutrition professionals in various areas is unquestionable. Mexico, Chile, Colombia and Perú showed a deficient nutritional workforce in their healthcare systems, but there is no information for Ecuador. There is a lack of job opportunities for Ecuadorian nutritionists, but a very high demand. It is imperative that nutritionists are included in every level of the healthcare system.

## Data Availability

The datasets used and/or analysed during the current study are available from the corresponding author on reasonable request.

## List of abbreviations

PR: Private
PU: Public
ODK: Open Data Kit
ICDA: International confederation of Dietetic Associations
ACOFANUD: Asociación Colombiana de Facultades de Nutrición y Dietética
ANNE: Asociación Nacional de Nutricionistas del Ecuador
MSP: Ministerio de Salud Pública del Ecuador
CNLAG: Ontario Clinical Nutrition Leaders Action Group
NHS: National Health Services

## References

[1] Ministerio de Salud Publica del Ecuador (MSP). ENCUESTA STEPS ECUADOR 2018 MSP, INEC, OPS/OMS Vigilancia de enfermedades no transmisibles y factores de riesgo [Internet]. Plataforma Gubernamental de Desarrollo Social. 2018. Available from: salud.gob.ec/wp-content/uploads/2020/10/INFORME-STEPS.pdf

[2] Instituto Nacional de Estadisticas y Censos INEC. Encuesta Nacional de Salud y Nutrición ENSANUT [Internet]. 2018 [cited 2022 May 16]. Available from: https://www.ecuadorencifras.gob.ec/documentos/web-inec/Estadisticas_Sociales/ENSANUT/ENSANUT_2018/PrincipalesresultadosENSANUT_2018.pdf

[3] Chee VA, Teran E, Hernandez I, Wright L, Izurieta R, Reina-Ortiz M et al. ‘Desculturización,’ urbanization, and nutrition transition among urban Kichwas Indigenous communities residing in the Andes highlands of Ecuador. Public Health. 2019 Nov1;176:21–8. 10.1016/j.puhe.2019.07.015 31679636

[4] De Andrade LOM, Filho AP, Solar O, Rígoli F, de Salazar LM, Serrate PCF (2015). Social determinants of health, universal health coverage, and sustainable development: case studies from latin american countries.

[5] Prowse RJ, Richmond SA, Carsley S, Manson H, Moloughney B. Strengthening public health nutrition: findings from a situational assessment to inform system-wide capacity building in Ontario, Canada. [cited 2023 Mar 4]; Available from: 10.1017/S1368980020001433 10.1017/S1368980020001433 PMC7557118 32618239

[6] Winklers MF. Standards of practice for the nutrition support dietitian: Importance and value to practitioners. J Am Diet Assoc. 1993 Oct 1;93(10):1113–8. 10.1016/0002-8223(93)91640-c 8409131

[7] Consejo de Educación Superior CES. Reglamento de Régimen Academico 2020 [Internet]. 2019. Available from: https://procuraduria.utpl.edu.ec/sitios/documentos/NormativasPublicas/Reglamento de Régimen Academico 2020.pdf

[8] MSP. Tipologia de Establecimientos de Salud por Niveles. Volume 2. Registro Oficial Suplemento; 2014.

[9] Pieniak Z, Żakowska-Biemans S, Kostyra E, Raats M. Sustainable healthy eating behaviour of young adults: towards a novel methodological approach. 2016 10.1186/s12889-016-3260-1 PMC4947369 27421759

[10] Von Elm E, Altman DG, Egger M, Pocock SJ, Gøtzsche PC, Vandenbroucke JP. The Strengthening the Reporting of Observational Studies in Epidemiology (STROBE) Statement: Guidelines for Reporting Observational Studies. PLoS Medicine | www [Internet]. 2007 [cited 2023 Mar 4];4(10):1623. Available from: http://www.epidem.com/10.1371/journal.pmed.0040296 PMC2020495 17941714

[11] Asociacion Mexicana de Miembros y Facultades y Escuelas de Nutrición AC (AMMFEN) (2010). Los empleadores de los nutriólogos en Mexico.

[12] Asociacion Mexicana de Miembros de Facultades y Escuelas de Nutricion AC. (AMMFEN). Los Nutriologos y la consulta privada. Mexico DF: Trillas; 2015. 91–156 p.

[13] Banco Central del Ecuador. Mercado laboral ecuatoriano. 2021.

[14] Instituto Nacional de Estadisticas y Censos INEC. Principales resultados de la Encuesta Nacional de Empleo, Desempleo y Subempleo - Anual 2021. 2021.

[15] Consejeria Presidencial para la equidad de la mujer. Día Internacional de Acción por la Salud de las Mujeres.

[16] Rosendo Zanga. Caracterización de los profesionales de la salud en Chile 2021 [Internet]. [cited 2023 Mar 2]. Available from: http://www.supersalud.gob.cl/portal/articles-20912_recurso_1.pdf

[17] Wyatt M, Dietrich L. The Dietitian Workforce in Canada: Meta-Analysis Report. 2012.

[18] Programa de las Naciones Unidas para el desarrollo (PNUD). MUJERES TRABAJADORAS EN EL SECTOR DE LA SALUD EN COLOMBIA [Internet]. 2022 [cited 2023 Mar 2]. Available from: https://www.undp.org/sites/g/files/zskgke326/files/2022-11/Documento%20de%20Trabajo%20Mujeres%20trabajadoras%20en%20el%20sector%20de%20la%20salud%20en%20Colombia.pdf

[19] Gheller BJF, Joy P, Lordly D (2018). A qualitative study exploring the experience of the male dietitian from student to professional. Can J Diet Pract Res.

[20] Joy P, Gheller B, Lordly D (2019). Men who are dietitians: deconstructing gender within the profession to inform recruitment. Can J Diet Pract Res.

[21] International Confederation of Dietetic Associations. Dietitians-nutritionists around the World Their Education and their Work. 2016.

[22] ACOFANUD. Perfil y competencias profesionales del Nutricionista Dietista en Colombia. Vol. 1, Comisión del ejercicio profecsonal de nutrción y dietética. 2013.

[23] MSP. Acuerdo-2016-N^o^-79. 2016.

[24] El Telegrafo. Por cada 20 camas debe haber un nutricionista [Internet]. 2018 [cited 2022 Feb 21]. Available from: https://www.eltelegrafo.com.ec/noticias/sociedad/6/salud-nutricion-tratamiento-ecuador

[25] Crovetto M (2015). Recurso Humano Nutricionista en hospitales publicos en Chile. Rev Med Chile.

[26] MINSA/DGSP. Norma Técnica de Salud de la Unidad Productora de Servicios de Salud de Nutrición y Dietética. 2013.

[27] MINSAL. normaalimentacionnutricion2005final. 2005.

[28] Clinical Nutritio Learders Action Group (CNLAG). Dietitian staffing levels In Ontario Hospitals A report from the Dietitians of Canada. 2018.

[29] National Quality Board. Supporting NHS providers to deliver the right staff, with the right skills, in the right place at the right time. Sage sustainable and productive staffing. NHS.uk. 2016.

[30] Perry S, Markham D, Evans K, Hoe T, Elliot K. Safe staffing, safe workload guidance.British Dietetic Assoiation. 2017;1–17.

[31] Instituto de Seguridad Social del Ecuador IESS. Remuneracion mensual por puesto [Internet]. 2020. Available from: https://www.iess.gob.ec/documents/10162/4440198/Remuneracion+mensual+por+puesto+parte+1

[32] Bureau of Labor Statistics US. Dietitians and Nutritionists : Occupational Outlook Handbook: : U.S. Bureau of Labor Statistics [Internet]. 2021 [cited 2022 Mar 9]. Available from: https://www.bls.gov/ooh/healthcare/dietitians-and-nutritionists.htm

[33] ESPOCH. Salud Pública celebra sus 49 Años y reconoce el trabajo de su comunidad - Escuela Superior Politécnica de Chimborazo [Internet]. [cited 2022 Feb 17]. Available from: https://www.espoch.edu.ec/index.php/component/k2/item/5202-salud-pública-celebra-sus-49-años-y-reconoce-el-trabajo-de-su-comunidad.html

